# An epithelial gene signature of trans-IL-6 signaling defines a subgroup of type 2-low asthma

**DOI:** 10.1186/s12931-023-02617-w

**Published:** 2023-12-07

**Authors:** Zaid W. El-Husseini, Dmitry Khalenkow, Andy Lan, Thys van der Molen, Chris Brightling, Alberto Papi, Klaus F. Rabe, Salman Siddiqui, Dave Singh, Monica Kraft, Bianca Beghe, Maarten van den Berge, Djoke van Gosliga, Martijn C. Nawijn, Stefan Rose-John, Gerard H. Koppelman, Reinoud Gosens

**Affiliations:** 1 Department of Pediatric Pulmonology and Pediatric Allergology, Beatrix Children’s Hospital, University of Groningen, University Medical Center Groningen, Groningen, The Netherlands; 2 University of Groningen, University Medical Center Groningen, Groningen Research Institute for Asthma and COPD (GRIAC), Groningen, The Netherlands; 3 Department of Molecular Pharmacology, Faculty of Science and Engineering, Groningen Research Institute of Pharmacy, University of Groningen, 9713 AV Groningen, The Netherlands; 4 Department of Infection, Immunity and Inflammation, Institute for Lung Health, University of Leicester, Leicester, UK; 5 Department of Respiratory Medicine, University of Ferrara, Ferrara, Italy; 6 Department of Medicine, Christian Albrechts University Kiel, Kiel and Lungen Clinic Grosshansdorf (Members of the German Center for Lung Research (DZL)), Grosshansdorf, Germany; 7 National Heart and Lung Institute, Imperial College and Imperial NIHR Biomedical Research Centre, London, UK; 8 Medicines Evaluation Unit, Manchester University NHS Foundation Hospital Trust, University of Manchester, Manchester, UK; 9 Department of Medicine, Icahn School of Medicine at Mount Sinai, New York, USA; 10 University of Modena and Reggio Emilia, AOU of Modena, Modena, Italy; 11 Department of Pulmonary Diseases, University of Groningen, University Medical Center Groningen, Groningen, The Netherlands; 12 Department of Pathology and Medical Biology, Experimental Pulmonary and Inflammatory Research (EXPIRE), University of Groningen, University Medical Centre Groningen, Groningen, The Netherlands; 13 Biochemical Institute, University of Kiel, Kiel, Germany

## Abstract

**Background:**

Asthma is stratified into type 2-high and type 2-low inflammatory phenotypes. Limited success has been achieved in developing drugs that target type 2-low inflammation. Previous studies have linked IL-6 signaling to severe asthma. IL-6 cooperates with soluble-IL-6Rα to activate cell signaling in airway epithelium.

**Objective:**

We sought to study the role of sIL-6Rα amplified IL-6 signaling in airway epithelium and to develop an IL-6+ sIL-6Rα gene signature that may be used to select asthma patients who potentially respond to anti-IL-6 therapy.

**Methods:**

Human airway epithelial cells were stimulated with combinations of IL-6, sIL-6Rα, and inhibitors, sgp130 (Olamkicept), and anti-IL-6R (Tocilizumab), to assess effects on pathway activation, epithelial barrier integrity, and gene expression. A gene signature was generated to identify IL-6 high patients using bronchial biopsies and nasal brushes.

**Results:**

Soluble-IL-6Rα amplified the activation of the IL-6 pathway, shown by the increase of STAT3 phosphorylation and stronger gene induction in airway epithelial cells compared to IL-6 alone. Olamkicept and Tocilizumab inhibited the effect of IL-6 + sIL-6Rα on gene expression. We developed an IL-6 + sIL-6Rα gene signature and observed enrichment of this signature in bronchial biopsies but not nasal brushes from asthma patients compared to healthy controls. An IL-6 + sIL-6Rα gene signature score was associated with lower levels of sputum eosinophils in asthma.

**Conclusion:**

sIL-6Rα amplifies IL-6 signaling in bronchial epithelial cells. Higher local airway IL-6 + sIL-6Rα signaling is observed in asthma patients with low sputum eosinophils.

**Supplementary Information:**

The online version contains supplementary material available at 10.1186/s12931-023-02617-w.

## Introduction

Asthma is a chronic inflammatory airway disease, clinically stratified into type 2-high and type 2-low. Type 2-high asthma is based on the presence of cytokines associated with type 2 inflammation (IL-4, IL-5, and IL-13), high levels of blood and sputum eosinophils, and elevated Fraction of exhaled Nitric Oxide. Type 2-low asthma is defined as absence of type 2 markers, but lacks biomarkers that could inform targeted treatment.

Limited success has been achieved in developing drugs that target type-2 low asthma [1]. Recently, we used genomics-guided drug target discovery to generate a list of potential novel targets that are implicated by SNPs associated with asthma [2]. One asthma SNP (rs4129267) in the *IL6R* gene is in high linkage disequilibrium with a non-synonymous SNP (rs2228145), which maps to the cleavage site of IL-6Rα. The risk allele for asthma (C) is associated with higher sIL-6Rα levels in serum [3–5]. Higher IL-6 levels are found in serum and sputum of asthma patients compared to non-asthmatic controls. Higher serum IL-6 levels also show association with metabolic syndrome and severe asthma [6–8]. IL-6 levels are also higher in the bronchoalveolar lavage fluid in type 2-low asthma compared to type 2-high asthma [9].

Interleukin-6 (IL-6) pathway activation depends on the interaction of IL-6 with the membrane-bound receptor IL-6Rα in combination with membrane-bound gp130 [10]. There are two forms of the receptor: the membrane-bound IL-6Rα and the soluble form of IL-6Rα (sIL-6Rα). sIL-6Rα is produced by either alternative splicing of the mRNA or by cleavage of the membrane-bound protein isoform of IL-6Rα by ADAM10 or ADAM17 [11, 12]. Typically, high expression levels of IL-6Rα are found on inflammatory cells such as macrophages and T cells, whereas gp130 is ubiquitously expressed. Accordingly, cleavage of IL-6Rα into sIL-6Rα represents a mechanism for cells not expressing IL-6Rα to allow IL-6 signaling. This is referred to as trans-IL-6 signaling whereas canonical IL-6 + IL-6Rα signaling is referred to as classic IL-6 signaling [13].

The link of the asthma susceptibility SNP in *IL6R* to sIL-6Rα levels prompted us to further investigate IL-6 trans-signaling. Moreover, a direct comparison of IL-6 classic and trans-signaling in airway epithelial cells and the effect of IL-6 pathway inhibitors has not been performed. Therefore, we sought to study the role of classic and trans IL-6 signaling in asthma in further detail and to use this information to develop an IL-6 gene signature that may be used to select potential patients for anti-IL-6 treatment. Specifically, we aimed to define the role of IL-6 classic versus IL-6 trans-signaling in airway epithelial cells and clarify their transcriptional and functional differences. Furthermore, we evaluated the association of an IL-6 signaling signature to different asthma-related phenotypes in bronchial biopsies and nasal brushes of asthma patients and controls.

## Methods (detailed information can be found in Additional file 1 and Additional file 2).

### Harvesting and culturing of hAECs

Primary human airway epithelial cells (hAECs) were collected from the trachea and main bronchus segments of lung transplant donors [14, 15].

For air–liquid interface (ALI) cultures, cells were subsequently seeded onto collagen-coated trans-well inserts. Subsequently, cells were exposed to air by removing the apical medium and refreshing the basolateral medium every other day to promote differentiation [14]. Cells were incubated at 37 °C; CO_2_ 5% for two weeks and stimulated with cytokines for 24 h following this 2-week differentiation period.

### Culturing BEAS-2B

Human bronchial epithelial cells (BEAS-2B) [16] were cultured in RPMI medium supplemented with 10% (v/v) FBS and 1% penicillin/streptomycin. Cells were incubated at 37 °C; CO_2_ 5%.

### Stimulation

Cells were stimulated with either a combination of 10 ng/ml of recombinant human IL-6 (R&D, 206-IL-010), 100 ng/ml of recombinant human IL-6Rα, 1000 ng/ml sgp130Fc (Olamkicept) [17, 18], or 1000 ng/ml Tocilizumab for 24 h (Additional file 1: Table S1). The concentrations of IL-6 and IL-6Rα were chosen based on Garbers et al. [19]. The concentrations of Olamkicept and Tocilizumab were based on Lin et al. and Lokau et al. respectively [20, 21].

### Protein extraction and Western blotting

BEAS-2B cultures were stimulated with IL-6, sIL-6R, Olamkicept, and Tocilizumab for 15 min. Samples were resolved using 10% SDS-PAGE, blotted onto Nitrocellulose membranes (Millipore), and probed with antibodies: STAT3, pSTAT3, α-tubulin, and GAPDH. Bands were visualized using the chemiluminescence substrate. The experiment used six biological replicates. The target protein levels were normalized to loading control (α-tubulin and GAPDH) and relatively compared to IL-6.

### xCELLigence

The xCELLigence®RTCA MP system was used. The 96-well E-plate was collagen-coated. The cellular impedance of the hAEC submerged culture was assessed every 15 min for a minimum of 24 h following stimulation. A Normalized Cell Index was defined as the mean cell index before stimulation.

### Transepithelial electrical resistance (TEER)

An Epithelial Voltammeter [22] (Millicell ERS-2 Voltohmmeter, Merck) was used to evaluate the epithelial barrier integrity in ALI culture by measuring the resistance (Ω) produced by the cultured cells. The measurements were taken before and after 24 h of stimulation.

### RNA extraction and RNA-seq analysis

Total RNA was isolated using NucleoSpin RNA purification kit (Macherey–Nagel 740955, Germany) according to the manufacturer’s guide. RNA-seq was conducted using the Illumina NovaSeq 6000 sequencer by GenomeScan (https://www.genomescan.nl/). The procedure included data quality control, adapter trimming, alignment of short reads, and feature counting. The human reference GRCh37.75 was used for alignment. Differential expression was assessed using *DESeq2* (v1.26.0) with a donor-adjusted linear model. Benjamini–Hochberg multiple testing correction was used for significance.

### IL-6 + sIL-6Rα signature generation

Genes were separated into quartiles depending on their baseline expression [23], then sorted depending on the log fold change within each quartile, and the top 5% taken of each set as potential candidates for a gene signature.

To validate the genes in the highest 4th expression quartile, a gene set enrichment analysis (GSEA) [24] was conducted and included the core enrichment genes. Normalized RNA-seq expression data of IL-6 + sIL-6Rα versus control generated in our study and IL-6 + sIL-6Rα versus the control condition derived from Jevnikar et al. [25] were used (Additional file 1: Table S2). Genes that were previously reported to be sensitive to stimulation with the type-2 cytokines IL-4 and IL-13 were excluded [25].

### Study population

To identify the asthma patient subgroup associated with the IL-6 + IL-6Rα pathway signature, bronchial biopsies from clinically well-characterized subjects were used. The subjects with current asthma were recruited from INDURAIN cohort (n = 77) [26], and non-asthma from NORM study (n = 66) [27]. The ATLANTIS study data is collected from nasal brushes and used to predict the signature enrichment in asthma patients (n = 362) against non-asthma (n = 58) [28].

## Results

### Effect of IL-6 and sIL-6Rα on airway epithelial cells

To investigate the effect of IL-6 and sIL-6Rα on airway epithelial cells, we assessed their influence on IL-6 signaling pathway activation, gene regulation, and cellular integrity.

**Fig. 1:**
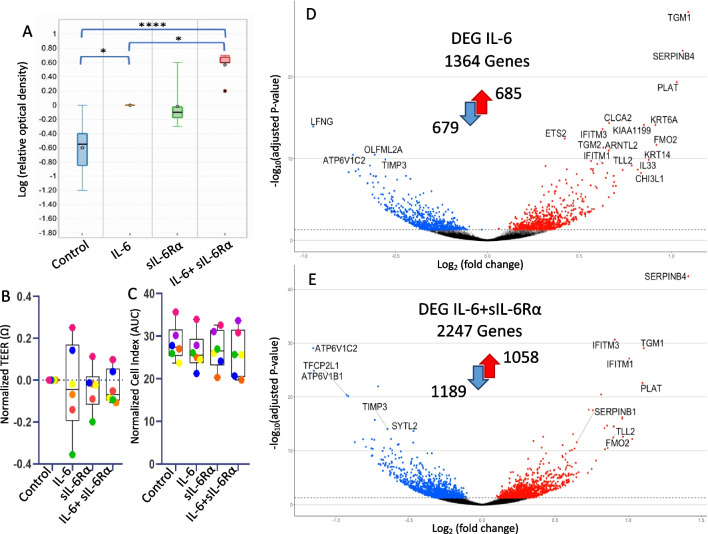
sIL-6R amplifies activation of the IL-6 pathway signaling and amplifies gene regulation in the airway epithelial cells. **A** Western blot analysis of BEAS-2B cells stimulated for 15 min solely or in combination with 10 ng/mL IL-6, 100 ng/mL sIL-6Rα, or a combination of both IL-6 and sIL-6Rα. Western blots were stained for pSTAT3 and quantified. Western blots represent six experiments. Statistically significant differences are indicated *p < 0.05; ****p < 0.0001. **B** Normalized TEER and **C**, xCELLigence show no effect of IL-6 and sIL-6Rα on the integrity of the epithelial barrier after and during 24 h of stimulation respectively. Differential gene expression analysis of **D** IL-6 vs. control and **E** IL-6 + sIL-6Rα vs control

To test the activation of the IL-6 + JAK/STAT3 signaling pathway, we stimulated BEAS-2B cells with IL-6 (10 ng/ml), sIL-6Rα (100 ng/ml), and the combination of both for 15 min. A significantly stronger STAT3 phosphorylation (pSTAT3) was detected after stimulation of BEAS-2B cells with IL-6 + sIL-6Rα complexes compared to IL-6 alone (Fig. 1A, Additional file 1: Table S3). Stimulation with sIL-6Rα alone showed a slight induction of STAT3 phosphorylation compared to the control.

To investigate the effect of IL-6, alone and in combination with sIL-6Rα on the integrity of the epithelial barrier, we performed TEER measurements after 24 h of stimulation of hAEC ALI cultures. The integrity of the epithelial barrier was not affected (Fig. 1B, Additional file 1: Table S4). We also performed a stimulation for 24 h on submerged hAEC to assess the growth properties of the cells in real-time using the xCELLigence. The outcome also showed that cell index (CI = impedance at time point n – impedance without cells/nominal impedance value) was not affected (Fig. 1C, Additional file 1: Table S5). The two experiments’ results suggest that 24 h of stimulation does not impair epithelial barrier integrity or cell viability, making these time points and concentrations suitable for further studies into gene regulation.

To identify the genes regulated by IL-6, IL-6Rα, or IL-6 + IL-6Rα, we stimulated hAEC ALI cultures for 24 h with these individual stimulations, followed by a differential gene expression analysis. In total, 1364 genes (*q* < 0.05) were found to be significantly differentially expressed between IL-6-stimulated cells vs. control (Fig. 1D, Additional file 1: Table S6). No genes were affected by sIL-6Rα alone and 2247 genes were significantly differentially expressed by IL-6 + sIL-6Rα vs. control (Fig. 1E, Additional file 1: Table S7). Highly induced genes were *SERPINB4, TGM1, PLAT*, and *ATP6V1C2*. Among these genes, 1058 were upregulated including asthma genes such as *IL4R, SMAD3, IL33, MUC5AC,* and *IL1RL1*. Two of the top five terms identified in network analysis (Additional file 1: Table S8) were the interleukin-33-mediated signaling pathway and negative regulation of T-helper 1 type immune response.

We next investigated the effect of sIL-6Rα on gene expression to study whether the addition of sIL-6Rα causes a unique molecular signature or merely amplifies IL-6 pathway signaling.

**Fig. 2:**
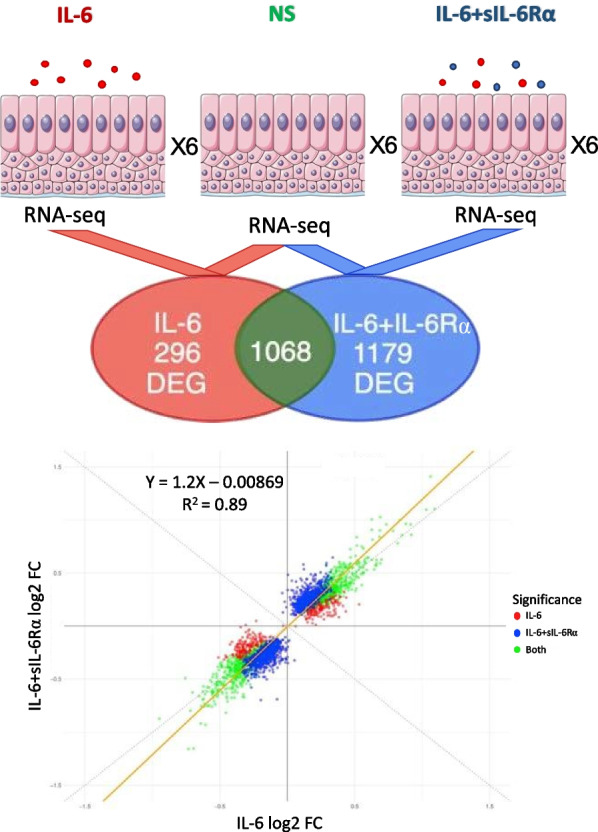
Scatter plot of the Log2 foldchanges of the differential gene expression analyses. Six fully differentiated hAEC were stimulated by IL-6, IL-6 + sIL-6Rα, or left unstimulated (control). Differential gene expression analyses were conducted using RNA-seq data of each of the stimulated conditions and compared against the control. Log2 foldchanges of the significant differentially expressed genes of both IL-6 and IL-6 + sIL-6Rα are shown in the scatterplot and directly compared to each other. The colors indicate in which analysis the gene was significant (q < 0.05). The dotted line represents a hypothetical correlation of y = x. The orange line is the linear fit of all the significant genes showing a trend towards the y-axis (y = 1.2x − 0.00869). NS; non-stimulated, DEG; differentially expressed genes

The DGE results show 1068 overlapping genes with differential expression in both the IL-6 vs. control and the IL-6 + sIL-6Rα vs. control comparisons (Fig. 2). We then plotted the fold change versus control of all genes, comparing the effect of IL-6 alone with that of IL-6 in combination with sIL-6Rα. As shown in Fig. 2, the differential expression levels of genes vs. the control are highly correlated among these two conditions with higher differential expression vs. the control when stimulated with IL-6 + sIL-6Rα. An interaction analysis showed no significant interaction between the presence or absence of sIL-6Ra and IL-6. These findings suggest that sIL-6Rα causes an amplification of the IL-6 pathway on the molecular level in airway epithelial cells leading to an amplification of IL-6 target gene expression without a distinct classic versus trans IL-6 signaling gene expression signature.

To confirm this contention further, we used 2 drugs, Tocilizumab and Olamkicept, that are used to inhibit IL-6 signaling through distinct mechanisms. Tocilizumab is an anti-IL-6R antibody that targets both, classic and trans IL-6 signaling. On the other hand, Olamkicept (sgp130Fc) chelates the IL-6 + sIL-6Rα complex preventing it from binding to membrane-bound gp130, thus inhibiting IL-6 trans-signaling specifically. The addition of Olamkicept to the cultures completely blocked the gene expression changes induced by IL-6 + sIL-6Rα, with 0 genes (q < 0.05) differentially expressed compared to the control. Similarly, the addition of Tocilizumab almost completely abrogated the gene expression changes induced by IL-6 + sIL-6Rα, leaving only 39 genes to be significantly differentially expressed compared to the control. At the pathway activation level, both Olamkicept and Tocilizumab showed a trend towards inhibition of STAT3 phosphorylation in BEAS-2B cells, however, it did not reach significance compared to the IL-6 + sIL-6Rα condition (Additional file 2: Fig. S2).

### IL-6 + sIL-6Rα gene signature in asthma

**Fig. 3:**
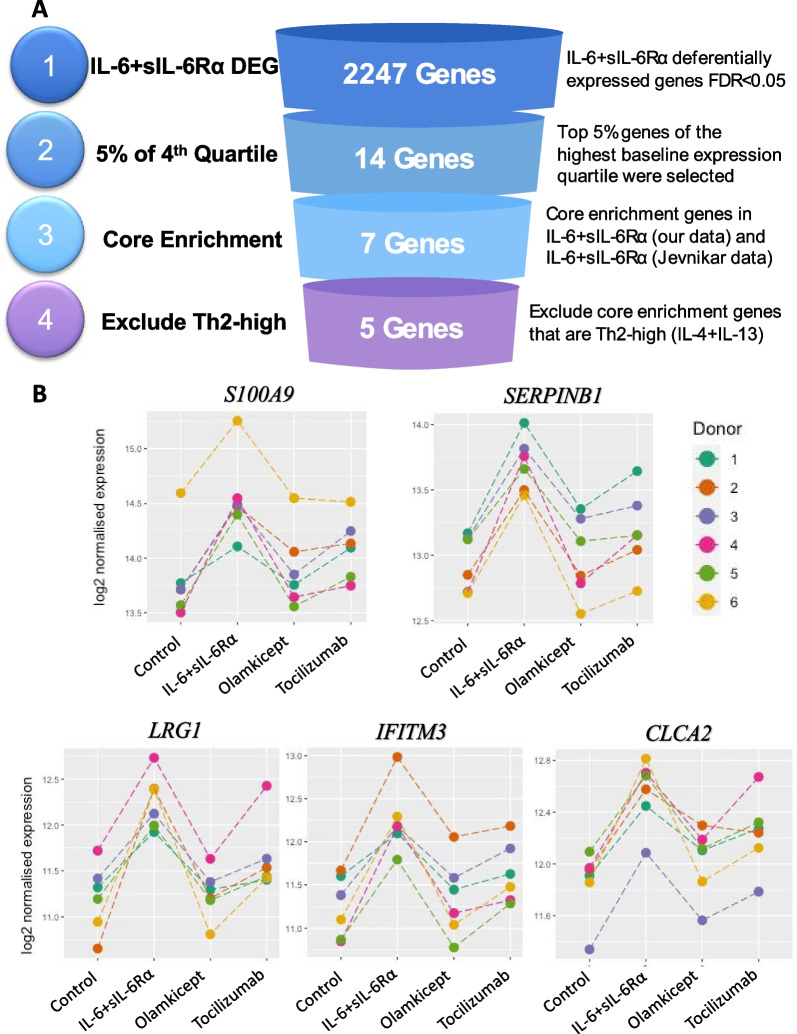
Generation of the IL-6 + sIL-6Rα Gene signature. The IL-6 + IL-6Rα Gene signature generation was generated from the significantly regulated genes in human airway epithelial cells by IL-6 + sIL-6Rα stimulation after passage through 3 filters. The core enrichment genes generated by GSEA [24], are genes that contribute to the leading-edge subset within the gene set. This is the subset of genes that contributes most to the enrichment result. B, The effect of Olamkicept and Tocilizumab on the IL-6 + sIL-6Rα gene signature expression in the presence of IL-6 + sIL-6Rα stimulation. DEG; differentially expressed genes, Th2; T helper type-2

A gene signature for IL-6 + sIL-6Rα was generated by using the quartile method [23], and the core enrichment genes from GSEA (Fig. 3A) which yielded an IL-6 + sIL-6Rα pathway signature of 5 genes (*S100A9, SERPINB1, LRG1, IFITM3, CLCA2*). We investigated the effect of Olamkicept and Tocilizumab on this signature in IL-6/IL-6R stimulated ALI cultured hAECs. All 5 genes were significantly downregulated by the addition of these drugs in all tested samples compared to IL-6 + sIL-6Rα stimulation (Fig. 3B, Additional file 1: Table S9). Olamkicept had a stronger inhibitory effect than Tocilizumab on the expression of these genes.

**Fig. 4:**
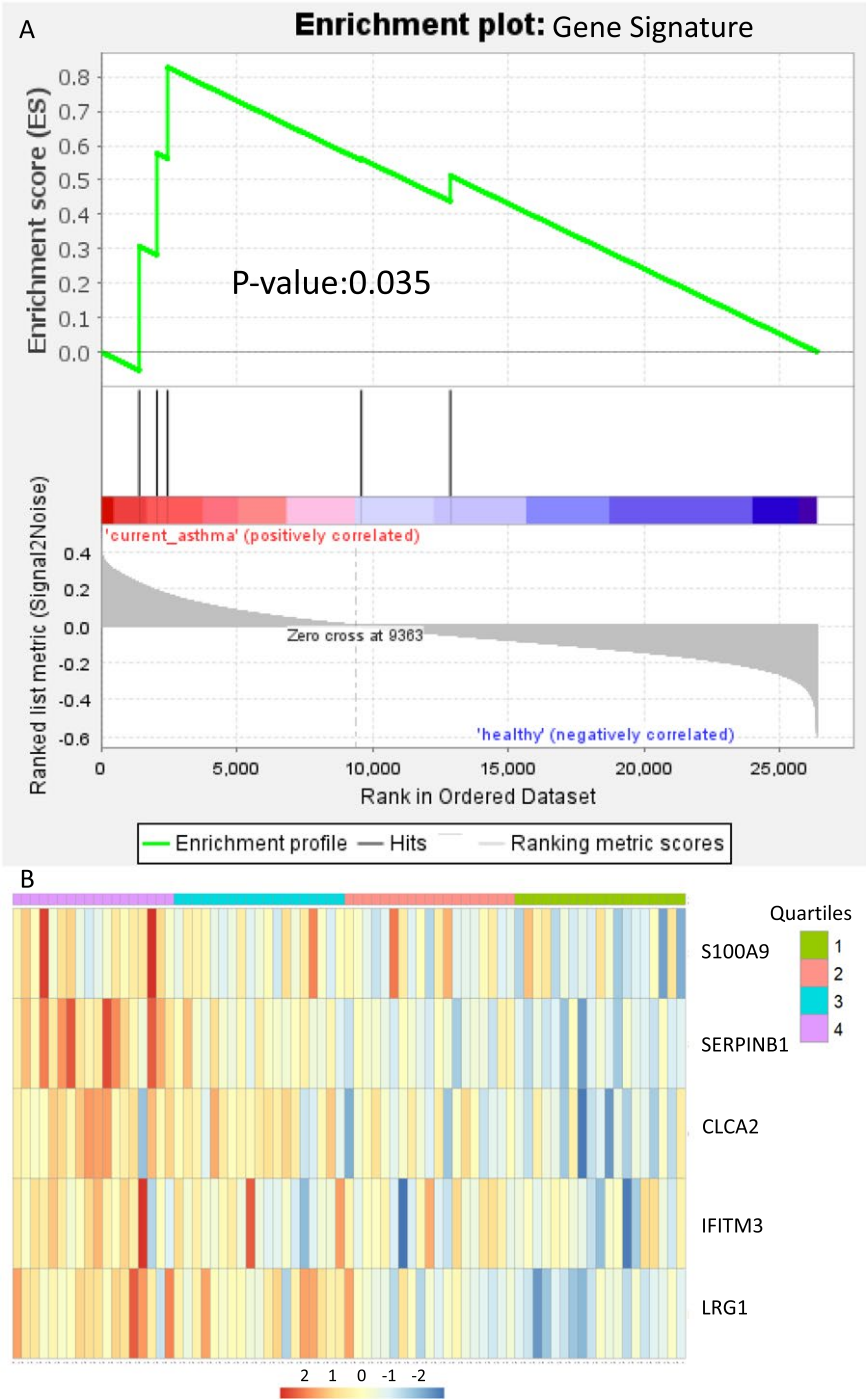
Expression of the IL-6 + sIL-6Rα gene signature in asthma. The IL-6 + sIL-6Rα gene signature was investigated in asthma patients and controls to study enrichment. **A** Enrichment plot of the Gene signature in asthma patients vs. controls, including the profile of the running enrichment score, and positions of gene-set members on the rank-ordered list. **B** patients were ranked based on the expression of the gene signature and divided into four quantiles. The IL-6 + sIL-6Rα high and IL-6 + sIL-6Rα low quartiles were used to inform the analyses reported in Table 1

The gene signature was then used as a potential approach to understand the significance of IL-6 signaling in human asthma (Additional file 1: Table S10), reasoning that high expression of this gene signature may reflect local epithelial IL-6 activation. Transcriptomic data from bronchial biopsies were used, which included two cohorts: Indurain (asthma patients, n = 77) [26], and NORM (non-asthmatic control subjects, n = 82) [27]. The IL-6 + sIL-6Rα gene signature score was significantly increased in transcriptomes from bronchial biopsies in asthma patients compared to healthy controls (Fig. 4A, Additional file 1: Table S11). However, no significant enrichment was detected in Atlantis cohort (asthma patients, n = 361 and controls, n = 57) (Additional file 1: Table S12), using nasal brushes as the source material [29]. We then stratified asthma patients based on the gene signature expression, into four quartiles to explore associations between the highest and lowest quartile with clinical features (Fig. 4B). Sputum eosinophil counts were found to be significantly lower in asthma patients with high expression of the IL-6 ^high^ gene signature compared to those with low expression of the gene signature in bronchial biopsies (Table 1).

**Table 1:** Comparison of clinical asthma phenotypes of patients with high expression of the IL-6 + sIL-6Rα gene signature versus low expression of gene signature

Asthma					
Phenotypes	High-Quartile		Low-Quartile		P-value
	Mean (± SD)	#N	Mean (± SD)	#N	
IL-6 + sIL-6Rα gene signature Z-score	0.79 ± 0.2	18	− 0.69 ± 0.32	19	2.2e−7
Age (y)	47 ± 9	18	49.16 ± 14	19	0.26
Male (N)	10	18	10	19	0.86
Smokers (N)	13	18	13	19	0.8
ICS users (N)	5	18	3	19	0.37
Body mass index (kg/m^2^)	28 ± 5.4	18	26.34 ± 5.17	19	0.15
Sputum eosinophils (10^6^)	0.009 ± 0.018	13	0.21 ± 0.35	15	0.0005*
Sputum eosinophils (%)	0.88 ± 1.2	13	6.3 ± 10.6	16	0.005*
Sputum neutrophils (10^6^)	0.77 ± 0.61	13	4.8 ± 12.1	15	0.053
Sputum neutrophils (%)	53 ± 23.6	13	57.6 ± 16.1	16	0.79
FEV_1_ (% predicted)	80.7 ± 21	18	83 ± 18.3	18	0.69
FEV_1_.FVC (%)	0.8 ± 0.02	18	0.8 ± 0.03	19	0.36
Total IgE (kU/L)	170 ± 303	11	240 ± 342	13	0.25
Alveolar concentration NO (ppb)	5.77 ± 2.71	13	7.46 ± 4.9	14	0.71
Bronchial flux of NO (nL/s)	0.59 ± 0.6	13	1.03 ± 0.73	14	0.48
CD3 T cells biopsy (cells/mm^2^)	74 ± 45	18	75.3 ± 55.8	19	0.94
CD4 T cells biopsy (cells/mm^2^)	21.85 ± 15.3	18	19.4 ± 16.5	19	0.48
CD8 T cells biopsy (cells/mm^2^)	29 ± 21.8	18	32.5 ± 23.2	19	0.53
CD68 macrophages biopsy (cells/mm^2^)	13.7 ± 11.7	18	15 ± 13.3	19	0.76
Blood eosinophils (%)	3.1 ± 2	17	4.3 ± 2	18	0.07
Blood basophils (%)	0.55 ± 0.65	17	0.8 ± 1	18	0.44
Blood lymphocytes (%)	35.49 ± 11.15	17	32.62 ± 9.8	18	0.39
Blood neutrophils (%)	56.48 ± 9.6	17	58.3 ± 10	18	0.54
Blood monocytes (%)	4.1 ± 3.5	17	3.843 ± 2	18	1

Data are presented as percentages, means ± SDs. For *P* values, the Mann–Whitney U test was used

*Statistically significant (P ≤ 0.05). *ICS* inhaled corticosteroids, *FEV*_*1*_ forced expiratory volume in 1 s, *FVC* forced vital capacity, *NO* nitric oxide

## Discussion

Previous genome-wide association studies along with clinical studies have shown an association between IL-6 signaling and asthma [25, 30, 31]. In this study, we investigated the effect of sIL-6Rα on cultured airway epithelial cells. The results of our work define the IL-6 and IL-6 + sIL-6Rα transcriptomic responses in airway epithelial cells and clarify both the nature of each of these responses and the question as to whether classic and trans-signaling in epithelial cells are distinct. This appears not to be the case; rather, sIL-6Rα amplifies IL-6 signaling without a unique trans-signaling response. Moreover, we generated a gene signature to predict the presence of active IL-6 + sIL-6Rα signaling in asthma patients and showed this to be enriched in asthma patients with low eosinophil counts.

Our results indicate that the IL-6 signaling pathway is activated by stimulating bronchial epithelial cells with IL-6 alone, which indicates that airway epithelial cells express functional IL-6Rα which is in line with a previous study [32]. We also examined the effect of IL-6 in combination with sIL-6Rα on the transcriptional level and found a large increase in the number and the effect size of up and down-regulated genes in comparison to the control, with no evidence of interaction between IL-6 and sIL-6Rα. These results indicate that the genes regulated by sIL-6Rα in combination with IL-6 are not distinct from the genes that were regulated by IL-6 alone. Therefore, a unique molecular IL-6 + sIL-6Rα trans-signaling pathway signature does not appear to exist in airway epithelial cells. This is in line with our findings that show that the presence of sIL-6Rα increases IL-6 pathway activation leading to increased phosphorylation of STAT3. These results match those mentioned in earlier studies by Jevnikar *et.al*. which showed an increase in STAT3 phosphorylation and the number of significantly regulated genes in the presence of sIL-6Rα in hAEC [25]. It should be noted though that the relationship between gene expression and function is not necessarily linear, and as such, the combination of IL-6 with sIL-6Rα may produce a distinct effect at the functional level.

A previous IL-6 trans gene signature was generated by Jevnikar *et.al* by stimulating bronchial epithelial cells in ALI cultures with IL-6 and sIL-6Rα [25]. Using the signature, a sub-group of asthma patients specifically characterized by high IL-6 trans-signaling was identified who had frequent exacerbations and submucosal infiltration of T cells and macrophages. In addition, this signature has been associated with neutrophil extracellular trap formation and *Haemophilus* infection in COPD [33]. Our method for the signature generation was different from the one presented in this paper. Jevnikar *et.al.* based it on the selection of genes forming a cluster in a subset of patients which was then referred to as IL-6TS–high. We also found that some of the selected genes had low baseline expression in cultured airway epithelial cells which made it difficult to be detected using qPCR (data not shown). Finally, some of the genes were highly co-expressed, such as *S100A8*, *S100A9*, and *S100A12*. To overcome the problem of selecting genes that have very low baseline expression we selected the top 5% DEG of the highest baseline-expression quartile [34]. After filtration based on GSEA analysis, five genes (*S100A9, SERPINB1, LRG1, IFITM3, CLCA2*) were selected to identify the activation of the IL-6 + sIL-6Rα pathway. The signature was carefully chosen to exclude genes activated by type-2 cytokine signaling such as IL-4 and IL-13. The signature was significantly enriched in bronchial biopsies from asthma patients in comparison with non-asthma patients (Fig. 4A), which demonstrates functional enrichment of the IL-6 + sIL-6Rα pathway in asthma. A previous study showed that gene expression networks in nasal brushes reflect gene expression networks in bronchial brushes [35]. Since we did not find an association with our IL-6 + sIL-6Rα signaling signature in nasal brushes obtained from asthma patients, this suggests a local role for IL-6 + sIL-6Rα signaling in the lower, but not in the upper airways. Alternatively, the IL-6 + sIL-6Rα gene expression signature may be dependent on cell types present in bronchial biopsies that are not present in epithelial brushes. This implicates that we cannot use this gene signature on nasal brushes to define IL-6-high asthma patients. We performed an additional exploratory analysis to understand the clinical characteristics of the patient subgroup that has high activation of the IL-6 + sIL-6Rα pathway based on the gene signature expression signature. We found that patients with high gene signature expression showed significantly lower sputum eosinophils compared to patients with low gene signature expression. Our results also show a trend toward lower sputum neutrophil numbers and blood eosinophil counts in patients characterized by high IL-6 + sIL-6Rα gene expression suggesting an association between IL-6 + sIL-6Rα signaling and paucigranulocytic or type-2 low asthma that warrants further investigation.

The genes associated with the IL-6 + sIL-6Rα gene expression signature (S100A9, SERPINB1, LRG1, IFITM3, and CLCA2) each play distinct roles in asthma. S100A9 is an intracellular cytoplasmic protein released following cellular damage that exhibits elevated levels in blood and sputum of neutrophilic asthma patients [36, 37], particularly in uncontrolled cases [38], contributing to innate immune responses and indicating its crucial role in neutrophilic airway inflammation [39]. Elevated S100A9 levels in severe asthma suggest involvement in disease progression [40]. SERPINB1, a proteinase/elastase inhibitor, protects against *Pseudomonas aeruginosa* lung infection in mice and may serve as a biomarker for cystic fibrosis-related inflammation [41, 42], with its role in asthma yet to be explored. Leucine-rich α-2 glycoprotein 1 (LRG1) is a secreted member of the family of leucine-rich repeat proteins, and is proposed as a biomarker for pneumonia and asthma-related airway inflammation [43, 44], potentially contributing to mucous overproduction through IL13-driven trans-differentiation of bronchial epithelial cells to goblet cells in asthma patients [44]. Interferon-induced transmembrane protein 3 (IFITM3), while unexplored in human allergic asthma, has been shown to drive and exacerbate Th2-cell responses in mouse studies [45], with genome studies linking IFITM2/3 variants to basophil counts and lung function [46, 47]. Chloride Channel Accessory 2 (CLCA2) has been implicated in airway diseases and contributes to secretory functions of airway epithelial cells in physiological and pathological processes, particularly in respiratory diseases like asthma and COPD [48].

In this study, two drugs, Olamkicept and Tocilizumab, were used to block the IL-6 pathway. Olamkicept (sgp130) is a drug that was generated with the objective of blocking the IL-6 trans-signaling pathway activated via sIL-6Rα by chelating the IL-6 + sIL-6Rα protein complex. On the other hand, Tocilizumab is a drug that binds and neutralizes IL-6Rα (both membrane-bound and soluble) in order to block the IL-6 pathway. At the gene expression level, both Olamkicept and Tocilizumab were able to effectively block all or most of the significantly regulated genes by IL-6 and sIL-6Rα, respectively. Thus, either drug was able to block IL-6 effects, at least at the transcriptional level. It is important to mention that the 2 drugs did not regulate any genes when present alone (data available upon request). Olamkicept tended to be more effective in reversing the gene expression signature than Tocilizumab, which could indicate more effective inhibition by the trans-signaling specific inhibition strategy. However, higher concentrations of Tocilizumab may have led to stronger inhibition of the effects; and we were not able to relate our gene expression effects to other, functional readouts of airway epithelial cells. Thus, the functional consequence of the slightly lower inhibition of the gene expression effect of Tocilizumab compared to Olamkicept remains to be established.

Previous studies indicated a pleiotropic role for IL-6 signaling in asthma. One of the factors that determine the role of IL-6 is the receptor form expressed by the target cell. The IL-6Rα membrane-bound receptor activates IL-6 classic signaling, which is known for its anti-inflammatory activities like promoting the regeneration of epithelial cells through transcription factor STAT3 [49], preventing epithelial cell apoptosis and inducing the hepatic acute phase response [50]. On the other hand, the soluble form of IL-6Rα (sIL-6Rα) activates IL-6 trans-signaling, which not only amplifies IL-6 signaling as we show in our paper but also transactivates IL-6 signaling in non-IL-6Rα expressing cells. This is known to lead to pro-inflammatory effects through the recruitment of mononuclear cells [51]. In addition, it negatively affects the restoration of tissue integrity and function by inhibiting TGF-β–induced Treg differentiation and inhibiting T-cell apoptosis [52, 53]. It has been reported that sIL-6Rα is elevated in the airways of asthma patients, and an association exists with severe asthma exacerbations in both children and adults [54, 55]. Although previous efforts attempted to target the IL-6 pathway in asthma, none targeted sIL-6Rα. The fact that tocilizumab and olamkicept in this study have comparable effects in blocking the induction of genes does not necessarily mean that these are clinically equally effective. It is possible that the selective blockade of sIL-6Rα prevents the recruitment of IL-6 transactivated cellular responses, whilst preserving the beneficial classic IL-6Rα driven signaling. Notably, Tocilizumab has shown its effectiveness in a range of inflammatory diseases in which IL-6 signaling plays a role, such as rheumatoid and juvenile idiopathic arthritis [56]. However, it wasn’t successful in suppressing the magnitude of the late asthmatic response in mild asthma patients, recorded between 3h and 7h after allergen challenge,  or of the early asthmatic response (recorded between 20 min and 2 h after allergen challenge) [57]. Olamkicept, on the other hand, selectively inhibits the trans-signaling pathway and is currently being developed for ulcerative colitis. In 2021, phase 2 clinical trials have been conducted to evaluate olamkicept in patients with active ulcerative colitis. The trials succeeded to demonstrate the clinical efficacy of olamkicept in the targeted patients which promises further development of the drug [58]. Our results suggest that a specific subgroup of IL-6 + sIL-6Rα high asthma patients exists. It would be an attractive hypothesis worth pursuing to investigate whether repurposing Olamkicept to this patient group may have clinical benefit.

### Supplementary Information

**Additional file 1: Table S1.** Different experimental conditions of stimulation or treatment with IL-6, sIL-6Rα, Olamkicept and Tocilizumab. **Table S2.**Gene set enrichment analysis of the 4th quartile top 5% IL-6+sIL-6Rα induced genes (core enrichment genes). **Table S3.**Quantitative measurement of western-blot band density relatively compared to IL-6. **Table S4.** Teer Measurement after normalization, compared to control condition. **Table S5.** Area under the curve of real-time cell impedance measurements using xCELLigence system after 24 hours of stimulation. **Table S6.** Differentially expressed Genes by IL-6 vs. control.**Table S7.** Differentially expressed Genes by IL-6+sIL-6Rα vs. control. **Table S8.** Functional enrichments in genes significantly upregulated by IL-6/IL-6Rα in Biological Process (Gene Ontology). **Table S9.** IL-6/IL6-Rα Gene signature expression in response to Olamkicipt and Tocilizumab. **Table S10.** Description of the Study population. **Table S11.** GSEA results summary in current Asthma phenotype in bronchial biopsies cohort. **Table S12.** GSEA results summary in current Asthma phenotype in nasal brush cohort. 

**Additional file 2: Extended Methods and Figure S1.** Western blot of the Pilot study of IL-6 stimulation showing 5 bands, the first is control in which nothing was added to the cells, bands 2-4 have different duration of IL-6 stimulation 1h, 30mins and 15 minutes, the last band is adding just the vehicle to the cells (PBS) to show disturbance doesn’t affect the outcome. The 15 minutes stimulation timepoint shows the optimal timepoint for the IL-6 pathway signaling activation in BEAS-2B cell-line. **Figure S2.**Western blot of all conditions.

## Data Availability

The datasets generated and/or analyzed during the current study are available from the corresponding author on reasonable request.

## Abbreviation

sIL-6Rα: Soluble form of IL-6Rα
hAECs: Human airway epithelial cells
ALI: Air liquid interface
RPMI: Roswell Park Memorial Institute medium
FBS: Fetal bovine serum
GAPDH: Glyceraldehyde 3-phosphate dehydrogenase
TEER: Trans-Epithelial Electrical Resistance
RNA: Ribonucleic acid
GSEA: Gene set enrichment analysis
pSTAT3: Phosphorylated STAT3
COPD: Chronic obstructive pulmonary disease
qPCR: Quantitative polymerase chain reaction
DEG: Differentially expressed genes
FEV1: Forced expiratory volume

## References

[1] Brusselle GG, Koppelman GH (2022). Biologic therapies for severe asthma. N Engl J Med.

[2] El-Husseini ZW, Gosens R, Dekker F, Koppelman GH (2020). The genetics of asthma and the promise of genomics-guided drug target discovery. Lancet Respir Med.

[3] Garbers C, Monhasery N, Aparicio-Siegmund S, Lokau J, Baran P, Nowell MA, Jones SA, Rose-John S, Scheller J (2014). The interleukin-6 receptor Asp358Ala single nucleotide polymorphism rs2228145 confers increased proteolytic conversion rates by ADAM proteases. Biochim Biophys Acta.

[4] Sarwar N, Butterworth AS, Freitag DF, Gregson J, Willeit P, Gorman DN, Gao P, Saleheen D, Rendon A, Nelson CP, Braund PS, Hall AS, Chasman DI, Tybjærg-Hansen A, Chambers JC, Benjamin EJ, Franks PW, Clarke R, Wilde AAM, Trip MD, Steri M, Witteman JCM, Qi L, van der Schoot CE, de Faire U, Erdmann J, Stringham HM, Koenig W, Rader DJ (2012). Interleukin-6 receptor pathways in coronary heart disease: a collaborative meta-analysis of 82 studies. Lancet (London, England).

[5] Swerdlow DI, Holmes MV, Kuchenbaecker KB, Engmann JEL, Shah T, Sofat R, Guo Y, Chung C, Peasey A, Pfister R, Mooijaart SP, Ireland HA, Leusink M, Langenberg C, Li K, Palmen J, Howard P, Cooper JA, Drenos F, Hardy J, Nalls MA, Li YR, Lowe G, Stewart M, Bielinski SJ, Peto J, Timpson NJ, Gallacher J, Dunlop M (2012). The interleukin-6 receptor as a target for prevention of coronary heart disease: a mendelian randomisation analysis. Lancet (London, England).

[6] Yokoyama A, Kohno N, Fujino S, Hamada H, Inoue Y, Fujioka S, Ishida S, Hiwada K (1995). Circulating interleukin-6 levels in patients with bronchial asthma. Am J Respir Crit Care Med.

[7] Neveu WA, Allard JL, Raymond DM, Bourassa LM, Burns SM, Bunn JY, Irvin CG, Kaminsky DA, Rincon M (2010). Elevation of IL-6 in the allergic asthmatic airway is independent of inflammation but associates with loss of central airway function. Respir Res.

[8] Peters MC, McGrath KW, Hawkins GA, Hastie AT, Levy BD, Israel E, Phillips BR, Mauger DT, Comhair SA, Erzurum SC, Johansson MW, Jarjour NN, Coverstone AM, Castro M, Holguin F, Wenzel SE, Woodruff PG, Bleecker ER, Fahy JV (2016). Plasma interleukin-6 concentrations, metabolic dysfunction, and asthma severity: a cross-sectional analysis of two cohorts. Lancet Respir Med.

[9] Virchow JC, Kroegel C, Walker C, Matthys H (1996). Inflammatory determinants of asthma severity: Mediator and cellular changes in bronchoalveolar lavage fluid of patients with severe asthma. J Allergy Clin Immunol.

[10] Kamimura D, Ishihara K, Hirano T (2003). IL-6 signal transduction and its physiological roles: the signal orchestration model. Rev Physiol Biochem Pharmacol..

[11] Matthews V, Schuster B, Schütze S, Bussmeyer I, Ludwig A, Hundhausen C, Sadowski T, Saftig P, Hartmann D, Kallen KJ, Rose-John S (2003). Cellular cholesterol depletion triggers shedding of the human interleukin-6 receptor by ADAM10 and ADAM17 (TACE). J Biol Chem.

[12] Horiuchi S, Koyanagiu Y, Zhouu Y, Miyamotou H, Tanakau Y, Waki M, Matsumoto A, Yamamotou M, Yamamotof N (1994). Soluble interleukin-6 receptors released from T cell or granulocyte/macrophage cell lines and human peripheral blood mononuclear cells are generated through an alternative splicing mechanism. Eur J Immunol.

[13] Garbers C, Heink S, Korn T, Rose-John S (2018). Interleukin-6: designing specific therapeutics for a complex cytokine. Nat Rev Drug Discov.

[14] Zuyderduyn S, Ninaber DK, Schrumpf JA, van Sterkenburg MAJA, Verhoosel RM, Prins FA, van Wetering S, Rabe KF, Hiemstra PS (2011). IL-4 and IL-13 exposure during mucociliary differentiation of bronchial epithelial cells increases antimicrobial activity and expression of antimicrobial peptides. Respir Res.

[15] Kistemaker LEM, Hiemstra PS, Bos IST, Bouwman S, Van Den Berge M, Hylkema MN, Meurs H, Kerstjens HAM, Gosens R (2015). Tiotropium attenuates IL-13-induced goblet cell metaplasia of human airway epithelial cells. Thorac Surg Clin.

[16] BEAS-2B Cell Line human | Sigma-Aldrich. <https://www.sigmaaldrich.com/NL/en/product/sigma/95102433?gclid=EAIaIQobChMIrOv8nu_A_gIVGsh3Ch3A2wb_EAAYASAAEgJeG_D_BwE&gclsrc=aw.ds>.

[17] Schreiber S, Aden K, Bernardes JP, Conrad C, Tran F, Höper H, Volk V, Mishra N, Blase JI, Nikolaus S, Bethge J, Kühbacher T, Röcken C, Chen M, Cottingham I, Petri N, Rasmussen BB, Lokau J, Lenk L, Garbers C, Feuerhake F, Rose-John S, Waetzig GH, Rosenstiel P (2021). Therapeutic Interleukin-6 trans-signaling inhibition by Olamkicept (sgp130Fc) in patients with active inflammatory bowel disease. Gastroenterology.

[18] Jostock T, Müllberg J, Özbek S, Atreya R, Blinn G, Voltz N, Fischer M, Neurath MF, Rose-John S (2001). Soluble gp130 is the natural inhibitor of soluble interleukin-6 receptor transsignaling responses. Eur J Biochem.

[19] Garbers C, Thaiss W, Jones GW, Waetzig GH, Lorenzen I, Guilhot F, Lissilaa R, Ferlin WG, Grötzinger J, Jones SA, Rose-John S, Scheller J (2011). Inhibition of classic signaling is a novel function of soluble glycoprotein 130 (sgp130), which is controlled by the ratio of interleukin 6 and soluble interleukin 6 receptor. J Biol Chem.

[20] Lin M, Rose-John S, Grötzinger J, Conrad U, Scheller J (2006). Functional expression of a biologically active fragment of soluble gp130 as an ELP-fusion protein in transgenic plants: purification via inverse transition cycling. Biochem J.

[21] Lokau J, Kleinegger F, Garbers Y, Waetzig GH, Grötzinger J, Rose-John S, Haybaeck J, Garbers C (2020). Tocilizumab does not block interleukin-6 (IL-6) signaling in murine cells. PLoS One..

[22] Merck. Millicell ® ERS-2 Electrical Resistance System User Guide. at <www.sigmaaldrich.com/deepweb/assets/sigmaaldrich/product/documents/407/335/00108103w-mk.pdf>.

[23] Jayalatha AKS, Badi Y, Ketelaar M, Hesse L, Kermani NZ, Meyer K, Van Den BM, Guryev V, Adcock I, Koppelman G, Nawijn M (2022). Gene signature for patient stratification: lessons learned from IL-33 stimulated TH2 genes. ERJ Open Res.

[24] Subramanian A, Tamayo P, Mootha VK, Mukherjee S, Ebert BL, Gillette MA, Paulovich A, Pomeroy SL, Golub TR, Lander ES, Mesirov JP (2005). Gene set enrichment analysis: a knowledge-based approach for interpreting genome-wide expression profiles. Proc Natl Acad Sci U S A.

[25] Jevnikar Z, Östling J, Ax E, Calvén J, Thörn K, Israelsson E, Öberg L, Singhania A, Lau LCK, Wilson SJ, Ward JA, Chauhan A, Sousa AR, De Meulder B, Loza MJ, Baribaud F, Sterk PJ, Chung KF, Sun K, Guo Y, Adcock IM, Payne D, Dahlen B, Chanez P, Shaw DE, Krug N, Hohlfeld JM, Sandström T, Djukanovic R (2019). Epithelial IL-6 trans-signaling defines a new asthma phenotype with increased airway inflammation. J Allergy Clin Immunol.

[26] Vermeulen CJ, Xu CJ, Vonk JM, ten Hacken NHT, Timens W, Heijink IH, Nawijn MC, Boekhoudt J, van Oosterhout AJ, Affleck K, Weckmann M, Koppelman GH, van den Berge M (2020). Differential DNA methylation in bronchial biopsies between persistent asthma and asthma in remission. Eur Respir J.

[27] Boudewijn IM, Faiz A, Steiling K, van der Wiel E, Telenga ED, Hoonhorst SJM, ten Hacken NHT, Brandsma CA, Kerstjens HAM, Timens W, Heijink IH, Jonker MR, de Bruin HG, Sebastiaan Vroegop J, Pasma HR, Boersma WG, Wielders P, van den Elshout F, Mansour K, Spira A, Lenburg ME, Guryev V, Postma DS, van den Berge M (2017). Nasal gene expression differentiates COPD from controls and overlaps bronchial gene expression. Respir Res..

[28] Kole TM, Vanden Berghe E, Kraft M, Vonk JM, Nawijn MC, Siddiqui S, Sun K, Fabbri LM, Rabe KF, Chung KF, Nicolini G, Papi A, Brightling C, Singh D, van der Molen T, Dahlén SE, Agusti A, Faner R, Wedzicha JA, Donaldson GC, Adcock IM, Lahousse L, Kerstjens HAM, van den Berge M, Badorrek P, Broeders M, Boersma WG, Chetta A, Cukier A (2023). Predictors and associations of the persistent airflow limitation phenotype in asthma: a post-hoc analysis of the ATLANTIS study. Lancet Respir Med.

[29] Postma DS, Brightling C, Baldi S, Van den Berge M, Fabbri LM, Gagnatelli A, Papi A, Van der Molen T, Rabe KF, Siddiqui S, Singh D, Nicolini G, Kraft M, Pizzichini E, Cukier A, Stelmach R, Olivenstein R, Zhang Q, Badorrek P, Gessner C, Scichilone N, Chetta A, Paggiaro P, Milleri S, D’Amato M, Spanevello A, Foschino MP, Boersma WG, Broeders M (2019). Exploring the relevance and extent of small airways dysfunction in asthma (ATLANTIS): baseline data from a prospective cohort study. Lancet Respir Med.

[30] Rincon M, Irvin CG (2012). Role of IL-6 in asthma and other inflammatory pulmonary diseases. Int J Biol Sci.

[31] Hawkins GA, Robinson MB, Hastie AT, Li X, Li H, Moore WC, Howard TD, Busse WW, Erzurum SC, Wenzel SE, Peters SP, Meyers DA, Bleecker ER (2012). The IL6R variation Asp358Ala is a potential modifier of lung function in subjects with asthma. J Allergy Clin Immunol.

[32] Francisco D, Wang Y, Conway M, Hurbon AN, Dy ABC, Addison KJ, Chu HW, Voelker DR, Ledford JG, Kraft M (2020). Surfactant Protein-A protects against IL-13-induced inflammation in asthma. J Immunol.

[33] Winslow S, Odqvist L, Diver S, Riise R, Abdillahi S, Wingren C, Lindmark H, Wellner A, Lundin S, Yrlid L, Ax E, Djukanovic R, Sridhar S, Higham A, Singh D, Southworth T, Brightling CE, Olsson HK, Jevnikar Z (2021). Multi-omics links IL-6 trans-signalling with neutrophil extracellular trap formation and Haemophilus infection in COPD. Eur Respir J.

[34] Jayalatha AS, Ketelaar M, Hesse L, Badi Y, Zounemat-Kermani N, Brouwer S, Dijk F, van den Berge M, Guryev V, Sayers I, Vonk J, Adcock I, Koppelman G, Nawijn M (2022). IL-33 induced gene expression in activated Th2 effector cells is dependent on IL-1RL1 haplotype and disease status. bioRxiv..

[35] Imkamp K, Bernal V, Grzegorzcyk M, Horvatovich P, Vermeulen CJ, Heijink IH, Guryev V, Kerstjens HAM, van den Berge M, Faiz A (2019). Gene network approach reveals co-expression patterns in nasal and bronchial epithelium. Sci Rep.

[36] Hur GY, Ye YM, Yang E, Park HS (2019). Serum potential biomarkers according to sputum inflammatory cell profiles in adult asthmatics. Korean J Intern Med.

[37] Lee TH, Chang HS, Bae DJ, Song HJ, Kim MS, Park JS, Jun JA, Lee SY, Uh ST, Kim SH, Park CS (2017). Role of S100A9 in the development of neutrophilic inflammation in asthmatics and in a murine model. Clin Immunol.

[38] Lee TH, Jang AS, Park JS, Kim TH, Choi YS, Shin HR, Park SW, Uh ST, Choi JS, Kim YH, Kim Y, Kim S, Chung IY, Jeong SH, Park CS (2013). Elevation of S100 calcium binding protein A9 in sputum of neutrophilic inflammation in severe uncontrolled asthma. Ann Allergy Asthma Immunol.

[39] Le PD, Yoon MG, Ban GY, Kim SH, Kim MA, Ye YM, Shin YS, Park HS (2016). Serum S100A8 and S100A9 enhance innate immune responses in the pathogenesis of Baker’s asthma. Int Arch Allergy Immunol.

[40] Quoc QL, Choi Y, Thi Bich TC, Yang EM, Shin YS, Park HS (2021). S100A9 in adult asthmatic patients: a biomarker for neutrophilic asthma. Exp Mol Med.

[41] Benarafa C, Priebe GP, Remold-O’Donnell E (2007). The neutrophil serine protease inhibitor serpinb1 preserves lung defense functions in Pseudomonas aeruginosa infection. J Exp Med.

[42] Cooley J, Sontag MK, Accurso FJ, Remold-O’Donnell E (2011). SerpinB1 in cystic fibrosis airway fluids: quantity, molecular form and mechanism of elastase inhibition. Eur Respir J.

[43] Ishida T, Kotani T, Serada S, Fujimoto M, Takeuchi T, Makino S, Naka T (2020). Correlation of increased serum leucine-rich α2-glycoprotein levels with disease prognosis, progression, and activity of interstitial pneumonia in patients with dermatomyositis: a retrospective study. PLoS One..

[44] Honda H, Fujimoto M, Miyamoto S, Ishikawa N, Serada S, Hattori N, Nomura S, Kohno N, Yokoyama A, Naka T (2016). Sputum leucine-rich alpha-2 glycoprotein as a marker of airway inflammation in asthma. PLoS One..

[45] Yánez DC, Sahni H, Ross S, Solanki A, Lau CI, Papaioannou E, Barbarulo A, Powell R, Lange UC, Adams DJ, Barenco M, Ono M, D’Acquisto F, Furmanski AL, Crompton T (2019). IFITM proteins drive type 2 T helper cell differentiation and exacerbate allergic airway inflammation. Eur J Immunol.

[46] Astle WJ, Elding H, Jiang T, Allen D, Ruklisa D, Mann AL, Mead D, Bouman H, Riveros-Mckay F, Kostadima MA, Lambourne JJ, Sivapalaratnam S, Downes K, Kundu K, Bomba L, Berentsen K, Bradley JR, Daugherty LC, Delaneau O, Freson K, Garner SF, Grassi L, Guerrero J, Haimel M, Janssen-Megens EM, Kaan A, Kamat M, Kim B, Mandoli A (2016). The allelic landscape of human blood cell trait variation and links to common complex disease. Cell.

[47] Kichaev G, Bhatia G, Loh PR, Gazal S, Burch K, Freund MK, Schoech A, Pasaniuc B, Price AL (2019). Leveraging polygenic functional enrichment to improve GWAS power. Am J Hum Genet.

[48] Sharma A, Ramena G, Yin Y, Premkumar L, Elble RC (2018). CLCA2 is a positive regulator of store-operated calcium entry and TMEM16A. PLoS One..

[49] Grivennikov S, Karin E, Terzic J, Mucida D, Yu GY, Vallabhapurapu S, Scheller J, Rose-John S, Cheroutre H, Eckmann L, Karin M (2009). IL-6 and STAT3 are required for survival of intestinal epithelial cells and development of colitis associated cancer. Cancer Cell.

[50] Barkhausen T, Tschernig T, Rosenstiel P, Van Griensven M, Vonberg RP, Dorsch M, Mueller-Heine A, Chalaris A, Scheller J, Rose-John S, Seegert D, Krettek C, Waetzig GH (2011). Selective blockade of interleukin-6 trans-signaling improves survival in a murine polymicrobial sepsis model. Crit Care Med.

[51] DeLeo FR (2007). Chemokines: attractive shedding. Blood.

[52] Jones GW, McLoughlin RM, Hammond VJ, Parker CR, Williams JD, Malhotra R, Scheller J, Williams AS, Rose-John S, Topley N, Jones SA (2010). Loss of CD4+ T cell IL-6R expression during inflammation underlines a role for IL-6 trans signaling in the local maintenance of Th17 cells. J Immunol.

[53] Dominitzki S, Fantini MC, Neufert C, Nikolaev A, Galle PR, Scheller J, Monteleone G, Rose-John S, Neurath MF, Becker C (2007). Cutting edge: trans-signaling via the soluble IL-6R abrogates the induction of FoxP3 in naive CD4+CD25 T cells. J Immunol.

[54] Doganci A, Eigenbrod T, Krug N, De SGT, Hausding M, Erpenbeck VJ, Haddad E-B, Schmitt E, Bopp T, Kallen K-J, Herz U, Schmitt S, Luft C, Hecht O, Hohlfeld JM, Ito H, Nishimoto N, Yoshizaki K, Kishimoto T, Rose-John S, Renz H, Neurath MF, Galle PR, Finotto S (2005). The IL-6R α chain controls lung CD4+CD25+ Treg development and function during allergic airway inflammation in vivo. J Clin Invest.

[55] Ghebre MA, Pang PH, Desai D, Hargadon B, Newby C, Woods J, Rapley L, Cohen SE, Herath A, Gaillard EA, May RD, Brightling CE (2019). Severe exacerbations in moderate-to-severe asthmatics are associated with increased pro-inflammatory and type 1 mediators in sputum and serum. BMC Pulm Med..

[56] Tanaka T, Narazaki M, Kishimoto T (2014). IL-6 in inflammation, immunity, and disease. Cold Spring Harb Perspect Biol.

[57] Revez JA, Bain LM, Watson RM, Towers M, Collins T, Killian KJ, O’Byrne PM, Gauvreau GM, Upham JW, Ferreira MAR (2019). Effects of interleukin-6 receptor blockade on allergen-induced airway responses in mild asthmatics. Clin Transl Immunol.

[58] Zhang S, Chen B, Wang B, Chen H, Li Y, Cao Q, Zhong J, Shieh MJ, Ran Z, Tang T, Yang M, Xu B, Wang Q, Liu Y, Ma L, Wang X, Zhang N, Zhang S, Guo W, Huang L, Schreiber S, Chen M (2023). Effect of induction therapy with olamkicept vs placebo on clinical response in patients with active ulcerative colitis: a randomized clinical trial. JAMA.

