# Supplementary material for: An epithelial gene signature of trans-IL-6 signaling defines a subgroup of type 2-low asthma
Source: Respir Res. 2023 Dec 7;24:308. doi: 10.1186/s12931-023-02617-w (PMC10704725; doi:10.1186/s12931-023-02617-w)

An epithelial gene signature of trans-IL-6 signaling
defines a subgroup of type-2 low asthma

Authors:

Zaid W. El-Husseini^1,2,3^, Dmitry Khalenkow^1,2^, Andy Lan^2,3^, Thys van der Molen^2^, Chris Brightling^4^, Alberto Papi^5^, Klaus F. Rabe^6^, Salman Siddiqui^7^, Dave Singh^8^, Monica Kraft^9^, Bianca Beghé^10^, Maarten van den Berge^2,11^, Djoke van Gosliga^1,2,12^, Martijn C. Nawijn^2,12^, Stefan Rose-John^13^, Gerard H. Koppelman^1,2^, Reinoud Gosens^2,3^

**Additional materials**

**Methods**

**Primary hAEC source and culture**

Primary human airway epithelial cells (hAECs) were collected from transplant lung tissue donors post-mortem, from residual tracheal and main stem bronchial tissue, within 1-8 h after a lung donation. The donors did not suffer from primary lung disease such as asthma or COPD and had a smoking history less than 20 packs per year according to Eurotransplant guidelines. The material was collected in carbogenated Krebs-Henseleit-buffer (composition in mM: 117.5 NaCl, 5.6 KCl, 1.18 MgSO4, 2.5 CaCl2, 1.28 NaH2PO4, 25 NaHCO3 and 5.5 glucose) (1, 2). Cells were cultured in Keratinocyte Serum Free Medium (KSFM, Invitrogen Cat. No. 17005034) with 2.5 ug/ml Human Recombinant Epidermal Growth Factor and 25 ug/ml Bovine Pituitary Extract (EGF/BPE, Invitrogen, 17005075), supplemented with isoproterenol 1 µM (Sigma-aldrich, I6504). Experiments in submerged cells were conducted by exposing cells to 10 ng/ml of recombinant human IL-6 (R&D 206-IL-010), 100 ng/ml of recombinant human sIL-6R alpha (R&D 227-SR-025/CF) 1000 ng/ml Olamkicept (The soluble gp130Fc protein), and 1000 ng/ml Tocilizumab (anti-human IL-6R antibody) which generated 10 conditions (supplementary table 1) (3, 4).

**ALI cultured cells:**

Primary hAEC from passage 1–2 were grown submerged on collagen-coated Transwell inserts until confluence, after which they were cultured at an air–liquid interface (ALI) and differentiated for 2 weeks in B/D medium (1:1), which is a mixture of DMEM [Gibco] and bronchial epithelial growth medium (BEGM) [Lonza], supplemented with 0.4% [w/v] bovine pituitary extract [BPE], 0.5 ng/ml epidermal growth factor [EGF], 5 µg/ml insulin, 10 µg/ml transferrin, 1 µM hydrocortisone, 6.5 ng/ml T3, 0.5 µg/ml epinephrine [all from Lonza], 15 ng/ml retinoic acid [Sigma chemical] 1.5 µg/ml bovine serum albumin [Sigma chemical] 0.5 mM sodium pyruvate [Gibco], 20 U/ml penicillin and 20 µg/ml streptomycin [Gibco] (2, 3). ALI cultures were maintained for 14 days at 37 °C in a humidified atmosphere of 5% CO_2_. After 14 days of culture, cells were exposed for 24h to 10 ng/ml of recombinant human IL-6 (R&D 206-IL-010), 100 ng/ml of recombinant human sIL-6Rα (R&D 227-SR-025/CF), 1000 ng/ml Olamkicept (The soluble gp130Fc protein), and 1000 ng/ml Tocilizumab (anti-human IL-6R antibody) that was added to the basal medium. Medium was refreshed three times per week. The apical side of the epithelial cells was washed with phosphate buffer saline (PBS) at the same time. 24h after stimulation, cells were collected for RNA analysis.

**BEAS-2B Cell culture and preparation:**

BEAS-2B cells were maintained in growth medium (RPMI 1640 (Gibco Cat. No. 52400025), 10% heat inactivated fetal bovine serum supplemented with 1% penicillin/streptomycin at 37°C in a humidified atmosphere of 5% CO_2_. BEAS-2B cells were passaged at a confluency of ~80-90%. Cells were washed twice with 1x PBS and were loosened enzymatically with Trypsin/EDTA (1X; Biowest Cat. No. L0930-100) incubating for ~5 minutes at 37 °C; CO2 5%. Trypsin was inactivated by adding 2n + 2 mL growth medium where n is volume of Trypsin. The cells were centrifuged at 500 Relative Centrifugal Force (RCF) removing leftover Trypsin. Cells were resuspended in growth medium and were passaged in T75 flasks seeded at 15.000-25.000 cells/cm^2^. BEAS-2B cells were seeded in 6 well culture plates at a density of 2.5 x 105/mL 48 h prior to stimulation and washed twice with PBS before and after synchronizing cellular status in S1 medium (growth medium with 1% FBS) for at least 24 h.

**Western blotting for pSTAT3:**

To determine IL-6 pathway activation, a pilot study was conducted on BEAS-2B cell-lines stimulated with IL-6 for either 15, 30, or 60 minutes. The densest band for pSTAT3 was observed after 15 mins (Supplementary Figure 1), which indicated the peak of the IL-6 pathway signaling activation. In subsequent experiments, cells were stimulated for 15 minutes with IL-6 (10 ng/ml), sIL-6Rα (100 ng/ml), IL-6+sIL-6Rα, IL-6+sIL-6Rα+Olamkicept (1000 ng/ml), and IL-6+sIL-6Rα+Tocilizumab (1000 ng/ml).

Subsequently, cells were washed twice with ice cold 1x PBS and lysed with ice cold lysis buffer (β-glycerolphosphate in RIPA buffer (NaCl 9 mg/mL, Tris-HCL 7.9 mg/mL (pH 7.4), 0.1 mL/mL 10% Igepal (NP-40), 0.025 mL/mL 10% Sodium Deoxycholate, 0.01 mL/mL 100mM EDTA), 1 mg/mL Aprotinin (Sigma Cat. No. A3428-10MG), 1 mg/mL Leupeptin, 1 mg/mL Pepstatin A (Sigma Cat. No. P4265-5MG), 5 ul/ml Sodium Orthovanadate (Na3VO4; Sigma Cat. No. S6508-10G), 1 ul/ml Sodium Fluoride (NaF) and transferred into pre-cooled tube and kept on ice. The sample was sonicated (Sonics, Vibra-Cell) for 1 minute with an interval of 5 sec. pulse followed by 5 sec. rest. Tubes were centrifuged at 12.000 RCF for 20 minutes at 4 oC and supernatant was collected. Protein concentration was measured using the Pierce BCA Protein Assay Kit (Thermofisher scientific, Cat. No. 23225).

After the protein extraction. The samples were separated by SDS page and transferred onto nitrocellulose membranes (Sigma, No. GE10600002). The membranes were blocked by ROTI or 5% BSA. The membranes with proteins were incubated overnight at 4°C with pSTAT3 (Cell Signaling Technology, 9145) 1:2000 dilution in 5% BSA dilution, STAT3 (Cell Signaling Technology, 9139) 1:1000 dilution in 5% milk dilution buffer (in TBST), α-tubulin (Cell Signaling Technology, 3873) 1:4000 dilution in ROTI (ROTH, No. A151.4), and GAPDH (Santa Cruz, sc-47724) 1:5000 dilution in ROTI (ROTH, No. A151.4). The membrane was then incubated with the secondary antibodies for 1 hour at room temperature. The secondary antibodies used were rabbit anti‐mouse (HRP) or goat anti‐rabbit (HRP) 1:3000 dilution (Cell Signaling Technology®, #7074). The experiment was replicated six times (figure 2). The signal originating from the immunoblots was visualized by addition of chemiluminescence substrate (Western Lightning Plus-ECL, Cat. No. NEL105001EA, Perkin Elmer) and detected and quantified using the G-box imaging system (Syngene, Cambridge, UK).

**Real-time cell impedance measurements**

Morphological changes in Primary human airway epithelial cells were measured using a

label-free, real-time cell impedance-based system; xCELLigence® RTCA MP system (ACEA

BIO). Here, we validated the xCELLigence system based on impedance measurement for real-time detection of cell death in hAEC (80,000 cells/well were seeded in collagen-coated 96-well E-plate (Agilent, 5232368001) containing gold microelectrodes fused to the bottom surface of the well plate. The impedance of the electron flow caused by cell attachment to the well (cell index) was measured every 30 minutes. The cells were seeded one day before treatment and the measurement of the cell index was performed for over than 24 hours following treatment. The cell index was normalized to 1, before the addition of various compounds.

**TEER:**

An Epithelial Voltammeter(5) (EVOM^2^) was used to evaluate the epithelial barrier integrity in ALI culture by measuring the resistance (Ω) produced by the cultured cells. The measurements were taken before stimulation and after 24 hours of stimulation twice. The mean of the two measurements at each time was taken. We also take the measurement with empty slot called RBLANK=180. The tissue resistance can be calculated by substitute Rblank from the measurement (R_TISSUE_(Ω) = R_TOTAL_ – R_BLANK_). The Teer reported can be calculated by multiplying RTISSUE(Ω) with the measurement area in cm^2^=1.12 (TEER_REPORTED_ = R_TISSUE_(Ω) × M_AREA_(cm^2^)). We normalized the measurements after stimulation to the measurement before stimulation e.g. Normalised measurement IL-6 #1= measurement IL-6 #1 after stimulation × (measurement IL-6 #1 before stimulation / measurement Control #1 before stimulation). Then compared the relative measurements across samples relative difference Normalized measurement IL-6 #1/ Normalized measurement Control #1.

**RNA-seq:**

Cells were lysed by adding a mixture of buffer RA1 and dithiothreitol (Sigma-Aldrich, 3483-12-3). Total RNA was extracted using the RNeasy mini kit (Qiagen, Venlo, the Netherlands) according to the manufacturer’s instructions. Equal amounts of total RNA were then reverse transcribed to cDNA. The yield of the isolated RNA was then measured using the NanoDrop 1000 spectrophotometer. RNA-seq was conducted using the Illumina NovaSeq 6000 sequencer by GenomeScan (https://www.genomescan.nl/). The procedure included data quality control, adapter trimming, alignment of short reads and feature counting. Checks for possible sample and barcode contamination were performed and a set of standard quality metrics for the raw dataset was determined using quality control tools (FstQC v0.34 and FastQA). Prior to alignment, the reads were trimmed for adapter sequences using Trimmomatic v0.30. To align the reads of each sample, the human reference GRCh37.75 was used. Data was analyzed with DESEQ2 in R studio V.3.6.3. Moreover, we investigated the gender distribution by examining gender-associated genes (*XIST*-gene and Y-chromosomal genes). To explore the influence of technical variation, a principal component analysis (PCA) was performed using R package *limma* (version 3.42.2) and *stats* (version 3.6.3).

**Differential gene expression analysis**

Differential expression was assessed using generalized linear model using a negative binomial distribution in the R package *DESeq2* (version 1.26.0). We corrected for donor by entering this as a factor in the linear model. Linear model was utilized to obtain statistically significant DEGs based on the difference in their expression values between different stimulations and treatments between samples within donor. We applied correction for multiple testing by controlling the false discovery rate at 0.05 using the Benjamini-Hochberg procedure without foldchange cut-off in *DESeq2*. Linear regression model was computed through *lmodel2* using the major axis (MA) method to investigate the correlation log2Foldchange between IL-6 *vs* unstimulated cells and IL-6TS *vs* unstimulated cells.

**Interaction analysis**

For interaction analysis we conducted the analysis on both DESeq2 and edgeR separately with the following design (Design= ~Donor+IL6+IL6R+IL6:IL6R). There were no significant genes regulated. Benjamini-Hochberg multiple testing correction was used for significance.

**Generating Gene signature**

In this study, the Quantiles method was used to separate the significantly differentially expressed genes into 4 quartiles depends on the Baseline-expression, then the top 5% of genes with the most fold change in the highest quartile were selected (6).

**Gene Set Enrichment Analysis**

1. **Cohort**

To identify the asthma patient subgroup associated with IL-6+IL-6Rα pathway signature, the bronchial biopsies from clinically well-characterized subjects were used (Supplementary table 10). The subjects with current asthma were recruited from INDURAIN cohort (n=77) (7), and non-asthma from NORM study (n=66) (8). The ATLANTIS study data is collected from nasal brushes and used to predict the signature enrichment in asthma patients (n=362) against non-asthma (n=58) (9). All samples were obtained and processed in the same center (University Medical Center Groningen, Groningen, the Netherlands) by the same team.

1. **Data analysis**

The GSEA analysis was used to investigate the association between IL-6+IL-6Rα pathway signature and clinical parameters of interest. GSEA analysis was done using Broad Institute’s Gene Set Enrichment Analysis (GSEA) v. 4.2.3 [build 10] software. Default parameters: *Signal2Noise* metric and 1000 gene set permutations, were used to determine significance of signature gene sets in subgroups of interest.

First, the enrichment of our and Jevnikar *etal* IL-6+IL-6Rα pathway signature was investigated using GSEA in patients’ groups with current asthma relative to non-asthma control. GSEA results shows the significant enrichment of Elhusseini *et al* IL-6+IL-6Rα pathway signature in patients with current asthma, while the Jevnikar *et al* signature was not significantly enriched. (Supplementary table 11).

Next, we checked whether it is necessary to correct for sex, smoking status and inhaled corticosteroids in subsequent post-hoc analysis within current asthma patients. The patients were divided into groups based on sex, smoking status and the usage of inhaled corticosteroids and GSEA analysis was carried out in resulted subgroups (Table 1). The GSEA results shows that the IL-6+IL-6Rα pathway signature is not associated with sex, smoking, nor ICS.

**Identification of patient’s subgroups associated with IL-6+IL-6Rα pathway signature using quantile geometric mean method**

The analysis was done in R as follows: first, the gene expression dataset was normalized using counts per million normalizations from edgeR package. Next, all non-signature genes were filtered out. The geometric mean based on signature genes expression was calculated for each patient. Normalize this into a z-score to obtain a signature score per patient. Subsequently, patients were divided into four quantiles, the 1^st^ and 4^th^ quantile with highest and lowest geometric means were used for identification of patient’s subgroups associated with IL-6+IL-6Rα pathway signature.

**Table:**

| Table S1. GSEA results summary of sex, ICS and smoking analysis in subset of patients with current asthma. | | | | | | |
| --- | --- | --- | --- | --- | --- | --- |
| IL-6+IL6Rα Signature | ES | NES | NOM p-val | FDR q-val | FWER p-val | RANK AT MAX |
| Sex | -0.56 | -1.02 | 0.451 | 0.646 | 0.495 | 1342 |
| ICS | -0.21 | -0.39 | 1 | 1 | 0.763 | 20808 |
| Smoking | -0.7 | -1.52 | 0.086 | 0.077 | 0.061 | 820 |
| ES, Enrichment Score; NES, Normalised Enrichment Score, FWER, Family-wise Error Rate | | | | | | |

**Figures:**


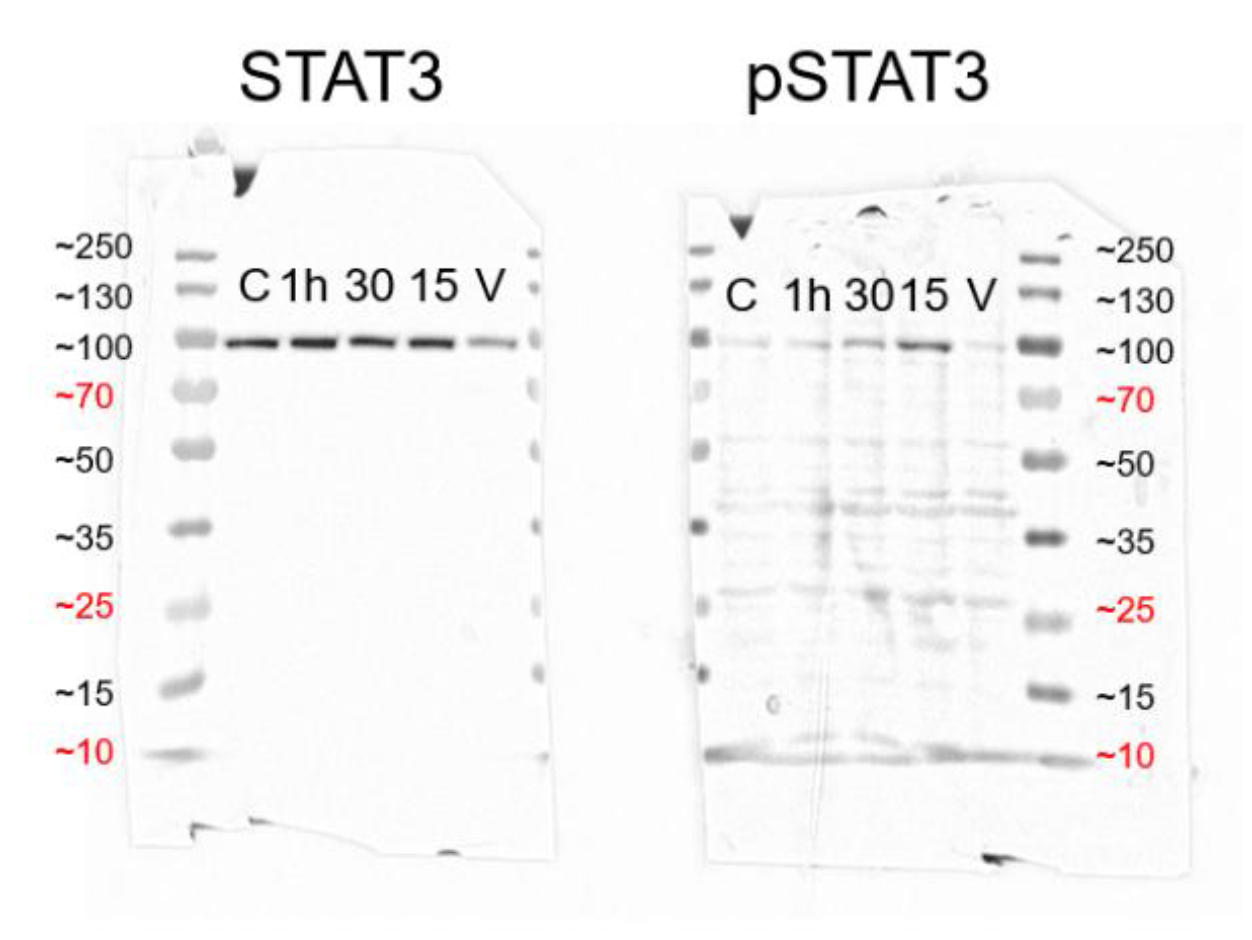


Figure S1: Western blot of the Pilot study of IL-6 stimulation showing 5 bands, the first is control in which nothing was added to the cells, bands 2-4 have different duration of IL-6 stimulation 1h, 30mins and 15 minutes, the last band is adding just the vehicle to the cells (PBS) to show disturbance doesn’t affect the outcome. The 15 minutes stimulation timepoint shows the optimal timepoint for the IL-6 pathway signaling activation in BEAS-2B cell-line.

Figure S2. Western blot of all conditions

In this western blot 10 conditions were selected with different combinations of IL-6 IL6R IL6IL6R Olamkicept and Tocilizumab stimulation to detect STAT3 phosphorylation and therefore activation of IL-6 signaling pathway. The IL6+IL6R stimulation shows the most activation of the IL-6 signaling pathway. Data was analyzed for statistical significance using one-way ANOVA. The padj-value indicating statistically significant differences between the mean values are defined as follows: *p<0.05, **p<0.01, ***p<0.001, ****p<0.0001. Statistical analyses were performed with GraphPad Prism 8 software.

References:

1. Rahmawati, S. F. *et al.* The effects of PGD2 and DK-PGD2 on features of airway remodeling in human airway epithelial cells and guinea pig lung slices. *Eur. Respir. J.* **56**, 1418 (2020).

2. Rahmawati, S. F. *et al.* Function-specific IL-17A and dexamethasone interactions in primary human airway epithelial cells. *Sci. Reports 2022 121* **12**, 1–13 (2022).

3. Zuyderduyn, S. *et al.* IL-4 and IL-13 exposure during mucociliary differentiation of bronchial epithelial cells increases antimicrobial activity and expression of antimicrobial peptides. *Respir. Res.* **12**, (2011).

4. Kistemaker, L. E. M. *et al.* Tiotropium attenuates IL-13-induced goblet cell metaplasia of human airway epithelial cells. *Thorax* **70**, 668–676 (2015).

5. Epithelial Voltohmmeter. https://www.wpi-europe.com/products/cell-and-tissue/teer-measurement/evom2.aspx.

6. Jayalatha, A. S. *et al.* IL-33 induced gene expression in activated Th2 effector cells is dependent on IL-1RL1 haplotype and disease status. *bioRxiv* 2022.10.20.513024 (2022) doi:10.1101/2022.10.20.513024.

7. Vermeulen, C. J. *et al.* Differential DNA methylation in bronchial biopsies between persistent asthma and asthma in remission. *Eur. Respir. J.* **55**, (2020).

8. Boudewijn, I. M. *et al.* Nasal gene expression differentiates COPD from controls and overlaps bronchial gene expression. *Respir. Res.* **18**, (2017).

9. Kole, T. M. *et al.* Predictors and associations of the persistent airflow limitation phenotype in asthma: a post-hoc analysis of the ATLANTIS study. *Lancet Respir. Med.* **11**, 55–64 (2023).

Western Blots of pSTAT3 and loading controls

**Sample1:**


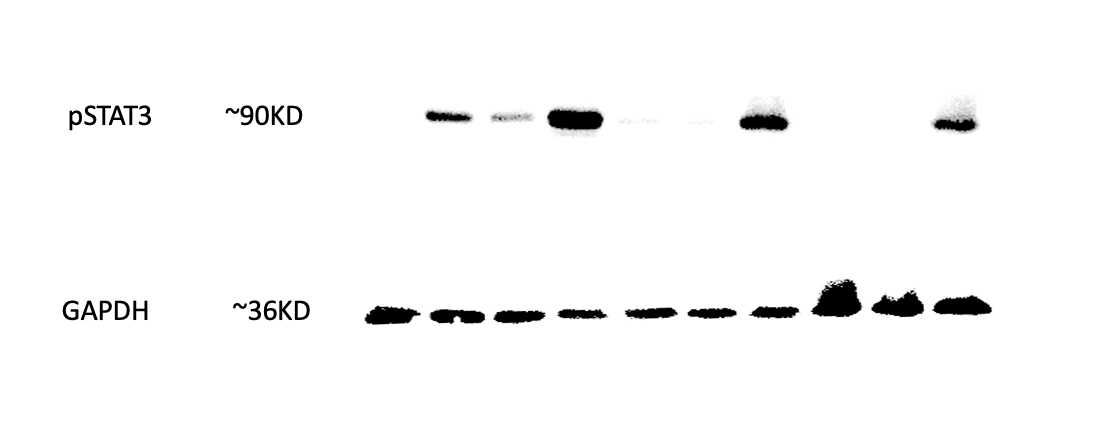


**Sample2:**


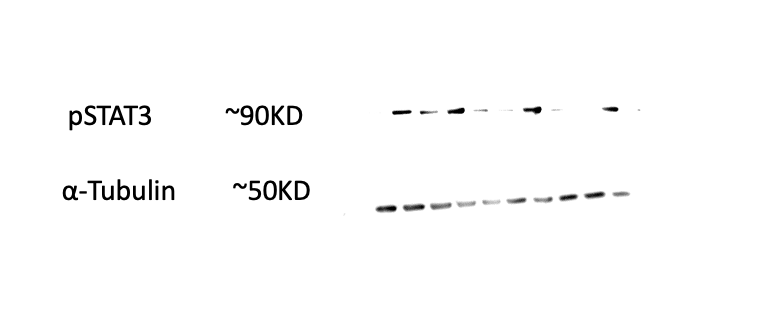


**Sample3:**


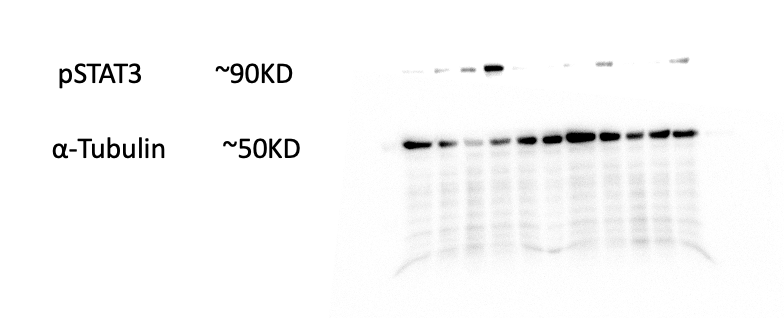


**Sample4:**


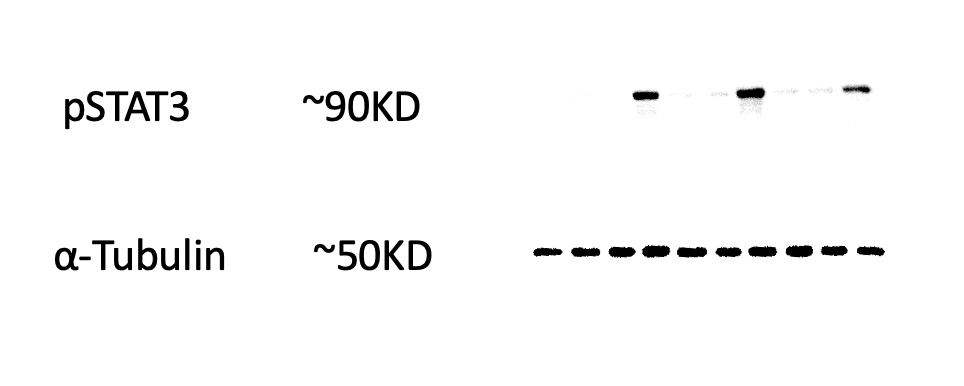


**Sample5:**


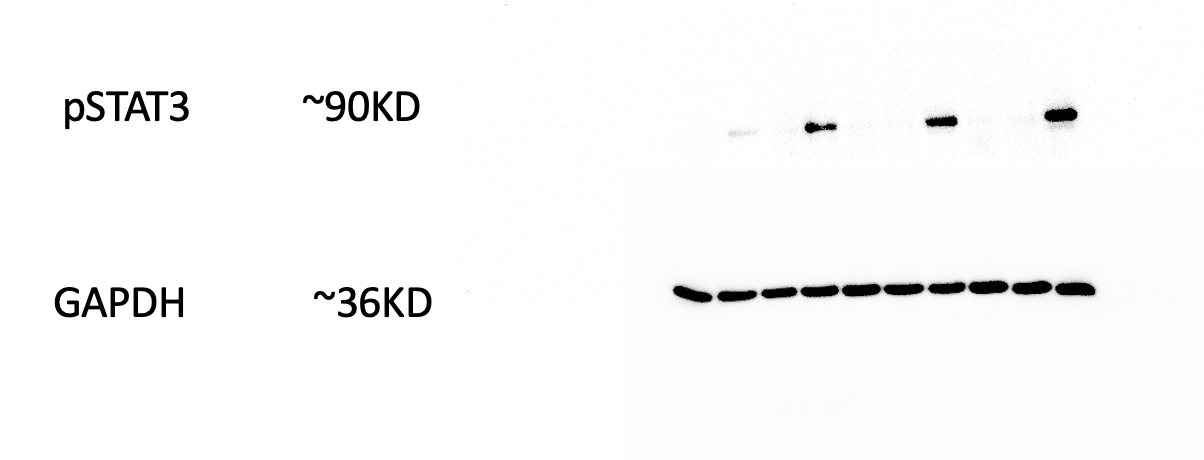


**Sample6:**


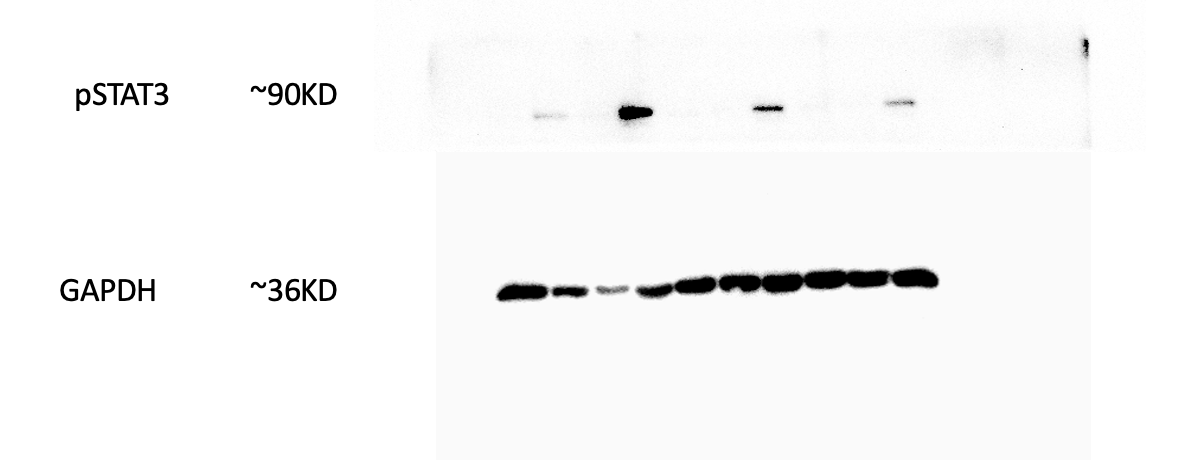

Supplement: Supplementary file 2 — Additional file 2: Extended Methods and Figure S1. Western blot of the Pilot study of IL-6 stimulation showing 5 bands, the first is control in which nothing was added to the cells, bands 2-4 have different duration of IL-6 stimulation 1h, 30mins and 15 minutes, the last band is adding just the vehicle to the cells (PBS) to show disturbance doesn’t affect the outcome. The 15 minutes stimulation timepoint shows the optimal timepoint for the IL-6 pathway signaling activation in BEAS-2B cell-line. Figure S2.Western blot of all conditions. [file 12931_2023_2617_MOESM2_ESM.docx]
